# Supplementary material for: Melittin from Bee Venom Encapsulating Electrospun Fibers as a Potential Antimicrobial Wound Dressing Patches for Skin Infections
Source: Pharmaceutics. 2022 Mar 28;14(4):725. doi: 10.3390/pharmaceutics14040725 (PMC9030956; doi:10.3390/pharmaceutics14040725)
Supplement: Supplementary file 1 [file pharmaceutics-14-00725-s001.zip › pharmaceutics-1658190-supplementary.pdf]

Supplementary Materials

Melittin from Bee Venom Encapsulating Electrospun Fibers as a Potential Antimicrobial Wound Dressing Patches for Skin Infections

Walaa S. Aburayan, Areej M. Alajmi, Ahmed J. Alfahad, Wijdan K. Alsharif , Abdullah A. Alshehri , Rayan Y. Booq, Samar A. Alsudir, Fatemah M. Alsulaihem, Haitham A. Bukhary , Moutaz Y. Badr, Essam J. Alyamani and Essam A. Tawfik

1. Melittin Quantification using Ultra-High-Performance Liquid Chromatography-MS/MS (UHPLC-MS/MS)

To quantify melittin fibers, a UHPLC-MS/MS method was developed, in which the UHPLC-UV, UHPLC-MS and calibration curve of melittin separation are shown in Figure S1, S2 and S3, respectively. The regression equation and correlation coefficient (R^2) were determined to be y = 4525.5x - 34681 and R^2= 0.997, respectively, indicating good linearity.

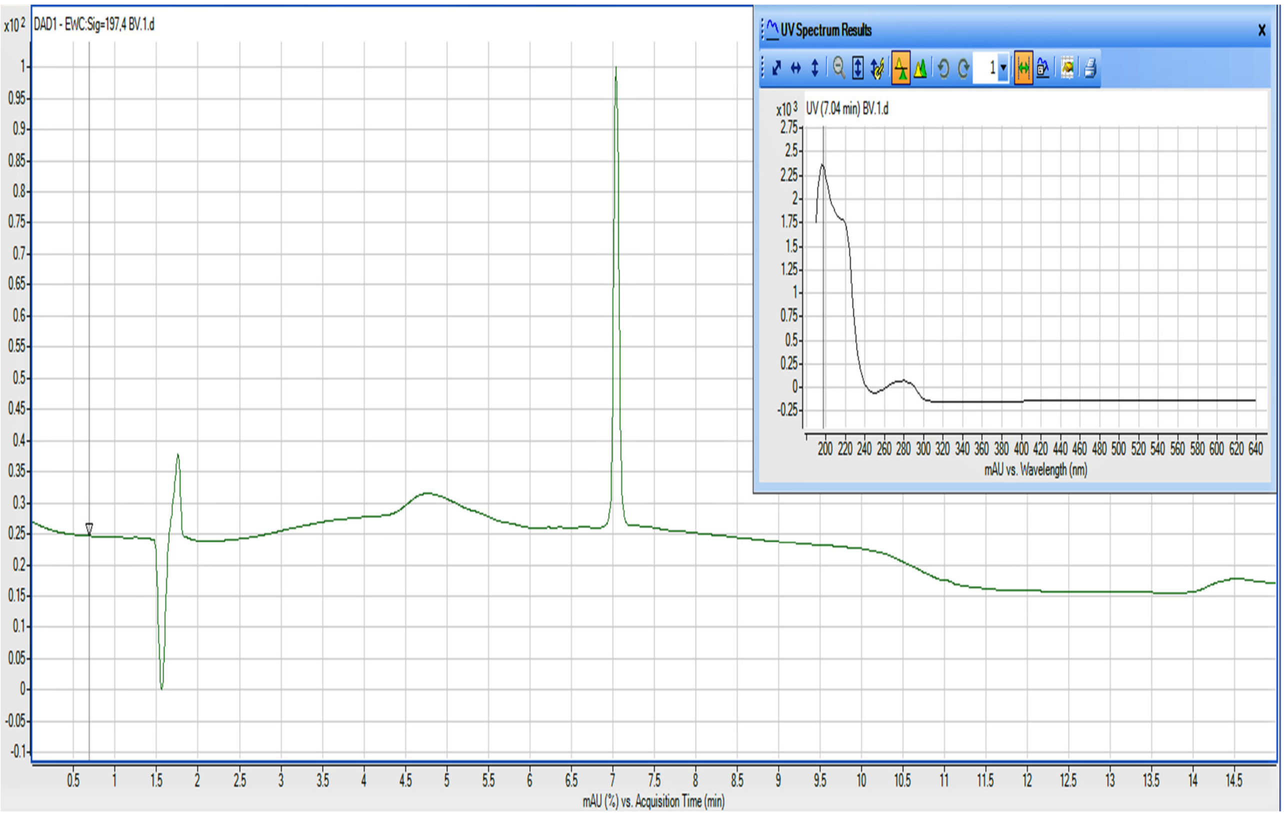

Figure S1. The UHPLC-UV chromatogram of Bee Venom shows the peak of melittin at R_t = 7.04 min and its UV spectrum on the top right.

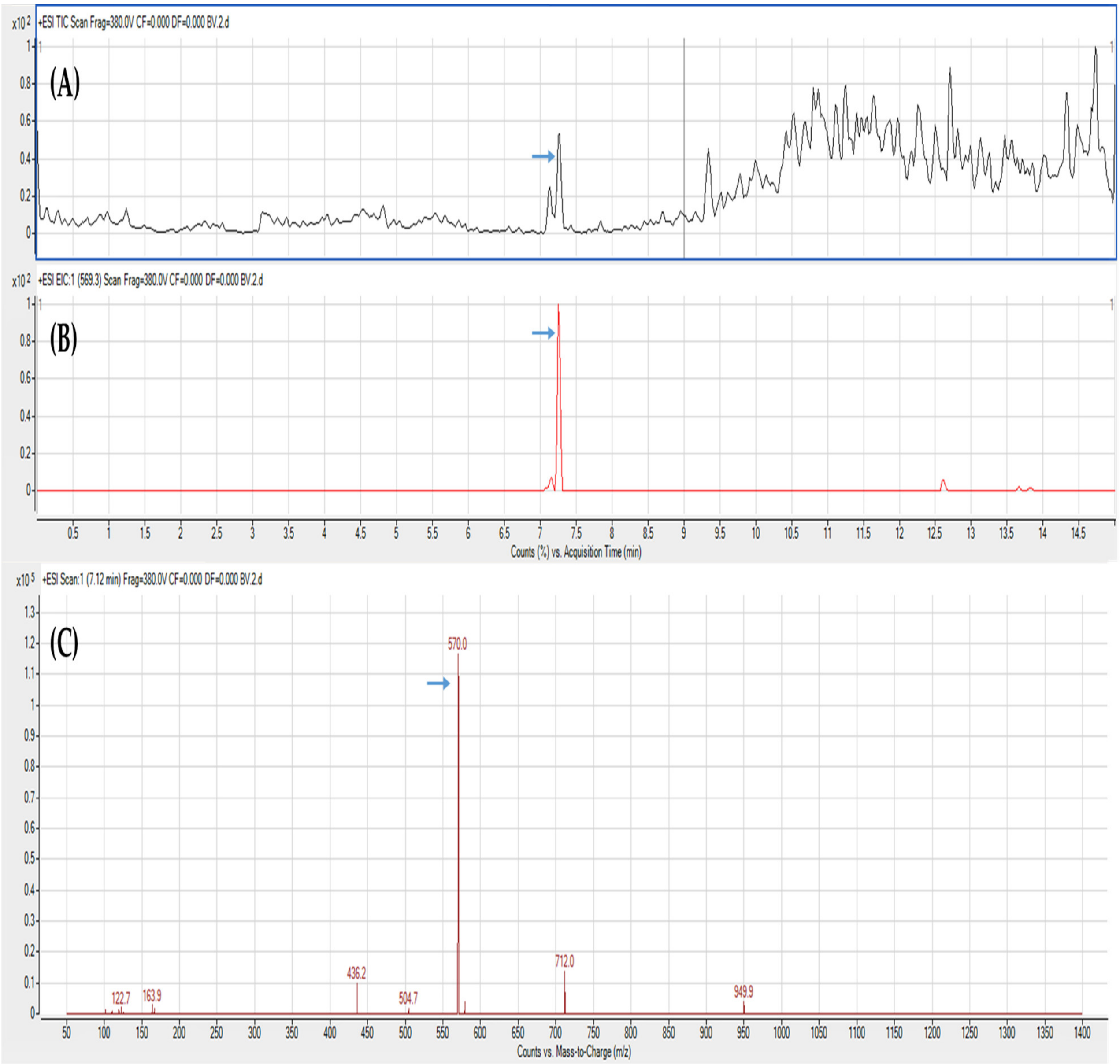

Figure S2. The UHPLC-MS chromatogram of Bee Venom shows **A**) The total ion current (TIC) chromatogram, **B**) The scan for 570 *m*/*z*, **C**) The mass spectrum of melittin.

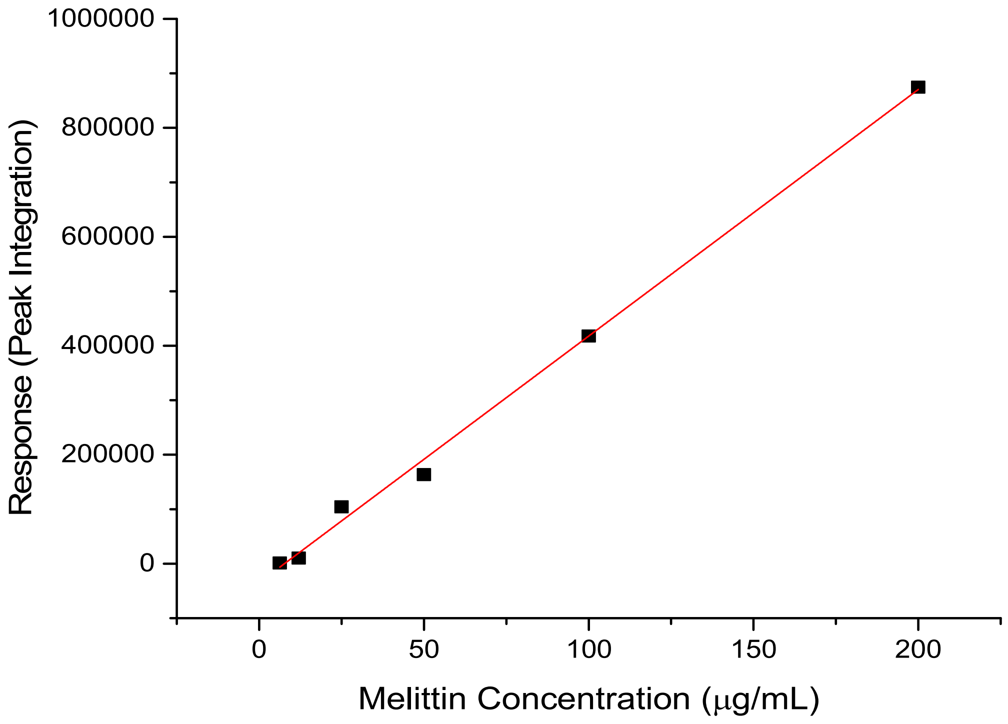

Figure S3. Melittin UHPLC-MS/MS calibration curve, showing good linearity at a concentration range of 200 to 6.25 μg/mL and R^2 of 0.997.

2. Minimum Inhibitory Concentration (MIC) of Melittin

A representative MIC result of melittin against *S. aureus* ATCC 977 is shown in Figure S4.

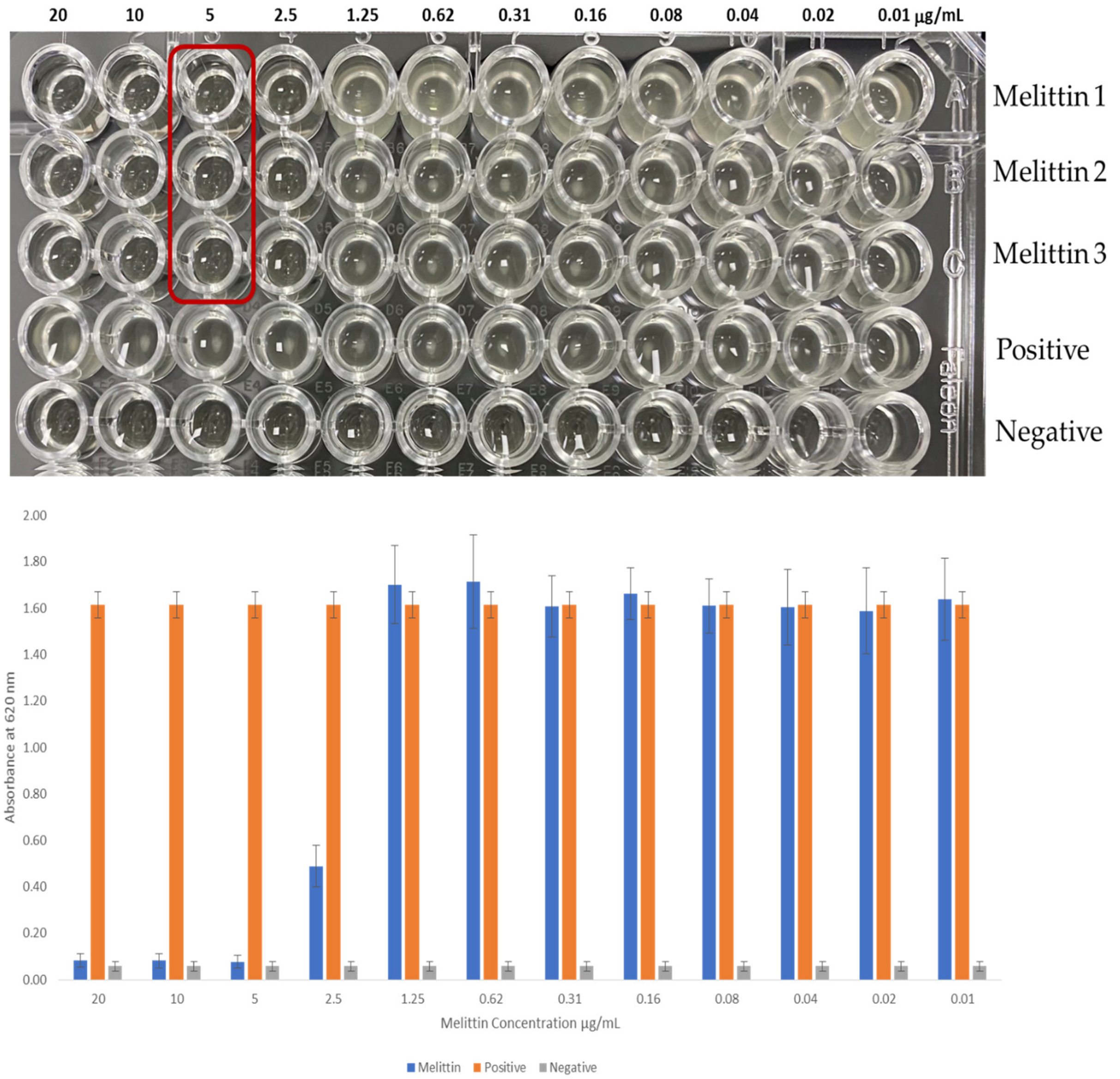

Figure S4. Melittin MIC against a representative bacterium strain *S. aureus* ATCC 977. The top image shows the MIC of melittin is 5 μg/mL, in which the bacterium growth was absent (lack of turbidity), while the bottom image shows melittin average concentrations against absorbance at 620 nm, where it showed no bacterium growth (no absorbance reading) at 5 μg/mL. The results are presented as average ± SD of independent triplicates.

3. Zone of Inhibition Assessment of Melittin-Loaded Fibers

A representative zone of inhibition result of melittin-loaded fibers (LF) compared to blank fibers (BF) and an equivalent amount of melittin discs (C), as experimental controls, against *S. aureus* ATCC 977 is demonstrated in Figure S5.

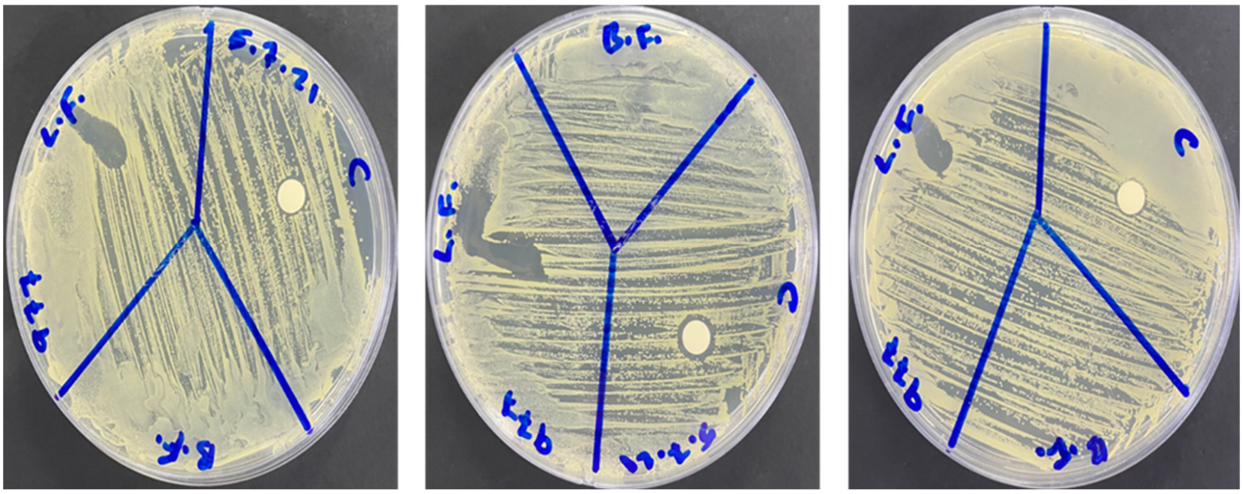

Figure S5: The zone of inhibition of melittin-loaded nanofibers against a representative bacterium strain *S. aureus* ATCC 977. Melittin-loaded nanofibers showed an inhibition zone of 8 ± 1 mm equivalent to the Melittin disc (control at a similar concentration), while the blank nanofibers (drug-free) showed no effect. The results are presented as the mean (± SD) of three replicates. C: Control, LF: Melittin nanofibers, BF: blank nanofibers.
